# The Reorganization of a Psychiatric Unit During COVID-19: A Reflection for Psychiatric Hospital Design

**DOI:** 10.1177/19375867221098982

**Published:** 2022-05-12

**Authors:** Jodi Sturge, Ferdi Starrenburg

**Affiliations:** 1 Adema Architecten, Groningen, the Netherlands; 2 Population Research Centre, University of Groningen, the Netherlands; 3 GGZ Friesland, Leeuwarden, the Netherlands

**Keywords:** COVID-19, transdisciplinary study, therapeutic landscapes, mental healthcare, psychiatry, evidence-based design

## Abstract

**Objective:**

The COVID-19 pandemic has impacted healthcare systems worldwide. Although this disease has primarily impacted general medicine intensive care units, other areas of healthcare including psychiatry were modified in response to corona measures to decrease the transmission of the disease. Reflecting on the modifications to the environment provides an opportunity to design psychiatric environments for future pandemics or other demands for healthcare.

**Background:**

The therapeutic environment of psychiatric wards was modified in Friesland, the Netherlands, in response to COVID-19. During this time, an interdisciplinary team met consistently to contribute to the preliminary design of a new psychiatric hospital.

**Methods:**

During the first 18 months of the pandemic, clinical reflections were made to describe the impact of COVID-19 on the psychiatric care environment. Architects have created a preliminary design of a new psychiatric hospital based on these reflections, monthly collaborative design discussions based on virtual mock-ups and evidence-based design based on theoretical concepts and research.

**Results and conclusions:**

This theoretical and reflective study describes how an inpatient psychiatric environment was restructured to manage infection during COVID-19. The therapeutic environment of the psychiatric ward and patient care changed drastically during COVID-19. The number of patients accessing care decreased, patient autonomy was restricted, and the function of designated behavioral support spaces changed to manage the risk of infection. However, these challenging times have provided an opportunity to reflect on theories and consider the design of new hospital environments that can be adapted in response to future pandemics or be restructured for different care functions.

## Background

Since December 2019, global healthcare systems have been exhausted due to the outbreak of coronavirus disease (COVID-19), caused by the severe acute respiratory syndrome coronavirus 2 (SARS-CoV-2) (Zhou et al., 2020). Although the main impact of this disease has been observed in general medicine intensive care units, other areas of healthcare have also been impacted by the transmission of the disease, including inpatient psychiatry patients and staff (Baumgart et al., 2021). Infection prevention and control practices emerged throughout inpatient psychiatry environments, which limited the autonomy of patients (Hernández-Calle et al., 2020), placed new pressure on staff (Knowles et al., 2020), and presented ethical challenges when patients refused testing or to comply with protective measures such as wearing masks (Fahed et al., 2020; Russ et al., 2020). Further, patient activities outside of wards were limited and personal and professional support (e.g., lawyers and judges) were restricted (Wasser et al., 2020).

Inpatient psychiatric wards consist of both voluntary and involuntary patients who require treatment for psychiatric illnesses. Patients are often admitted for extended periods, and repeated readmissions are common. Compared to other hospital environments, inpatient psychiatric wards tend to have higher patient autonomy. The environment is built around the principles of safety and protection from self-harm rather than infection control (Angelino et al., 2020; Lundin, 2021). The treatment environment often consists of physical and social features that support healing and have positive impact on staff and patients, such as the spatial qualities of inpatient care, such as access to private rooms, open nursing stations, and access to nature (Chrysikou, 2014; Molin et al., 2021; Shepley & Pasha, 2017; Shepley et al., 2016; Wood et al., 2013). Described as “spaces of transition,” the psychiatric environment provides stability, space for social interaction, and skill development for patients to successfully transition back to the community (Chrysikou, 2019; Wood et al., 2013). The design of psychiatric environments is transforming from a stale institutional environment to healing into therapeutic environments, focusing on design features that result in less aggression and a reduction of the use of coercion (e.g., seclusion or restraint) on the wards (Oostermeijer et al., 2021; Papoulias et al., 2014; Seppänen et al., 2018; Shepley & Pasha, 2017; Shepley et al., 2016; Ulrich et al., 2018). Based on Ulrich’s work, there has been a recent movement to design psychiatric environments based on healing architecture (Lundin, 2021). Although this terminology is familiar to Scandinavian countries (Frandsen et al., 2012; Simonsen & Duff, 2020, 2021), a recent review suggests no standard definition of healing architecture (Simonsen et al., 2022). Researchers have provided the examples of how the built psychiatric care environment is a coproducer of care (Simonsen & Duff, 2020, 2021; Wood et al., 2013, 2015). This work reflects health geography studies that have informed the development of models of care (Curtis, 2007; Curtis et al., 2013) that describe the psychiatric ward environment as a therapeutic landscape. In this context, it is relevant to differentiate the theory of therapeutic landscapes from “landscape architecture,” which refers to the practice of designing and creating physical landscapes. Therapeutic landscape is a theoretical conceptual framework that focuses on “how the healing process works itself out in places (or situations, locales, settings and milieus)” (Gesler, 1992, p. 743). There have been several studies exploring how well-being is influenced by green and blue therapeutic landscapes (Bell et al., 2014, 2017, 2018) and how built environment spaces, such as libraries and the world-renowned Maggie centre care model, are experienced and constructed to be therapeutic landscapes (Brewster, 2014; Butterfield & Martin, 2016). Further, the therapeutic landscape concept has been used to describe psychiatric environments with interest in how the design of psychiatric environments can support or constrain relations between staff, patients, and family (Curtis et al., 2007; Gesler et al., 2004; Wood et al., 2013, 2015). Until recently, there have been limited theoretical approaches or principles for designing the physical environment of psychiatric and mental healthcare facilities (Liddicoat et al., 2020; Ulrich et al., 2018).

The purpose of this case study is to describe the impact of COVID-19 pandemic on the therapeutic landscape of an inpatient psychiatric care environment in Friesland, the Netherlands, and link these reflections to the design of a new psychiatric environment. While other narrative reviews have explored the impact of COVID-19 on the psychiatric care environment (Angelino et al., 2020; Naarding et al., 2020), this interprofessional study is unique where it is based on 18 months of clinical reflections and an integrated theoretical reflection to design. The first author is a health geographer with research experience related to well-being and public space and the second author is a psychiatrist working in high intensive care in psychiatry. Together, we bring different perspectives to the psychiatric environment, viewing it as both a therapeutic landscape and a clinical space. This study has administrative approval from the geestelijke gezondheidszorg (GGZ) Friesland. Ethical review and approval were waived for this study, where the study did not involve the participation of human subjects. This study is based on 18 months of clinical descriptions (e.g., number of admissions) and reflections on the psychiatric environment. There is no identifying information of the patients or staff during the period of time of this study making it not possible to link a description to an individual case.

## Method

### Study Context

**Figure 1.**
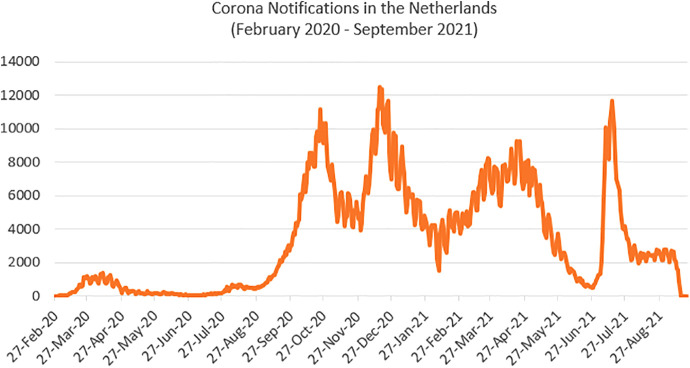
COVID-19 infection notifications in the Netherlands. *Source.* Derived from the National Institute for Public Health and the Environment (RIVM) Open Source Data. Accessed on September 21, 2021, from https://www.rivm.nl/coronavirus-covid-19/grafieken

On February 27, 2020, the first patient with COVID-19 in the Netherlands was confirmed. Three weeks later, the Dutch government implemented strict social distancing policies and measures to mitigate the spread of COVID-19 (Dinmohamed et al., 2020). By mid-March, most Dutch people were confined to their homes due to a nationwide lockdown (Naarding et al., 2020). Since the beginning of the pandemic in the Netherlands, there were several peaks in infections, which made it challenging to relax national government corona measures (Figure 1).

In the Netherlands, mental healthcare is delivered through regional network associations known as the GGZ. This case study is based on a collaborative design phase of a new high intensive inpatient psychiatry environment in the province of Friesland, located in the northern part of the Netherlands. High intensive inpatient psychiatry services in the province are provided across two sites; 28 beds in Leeuwarden and 17 beds in Heerenveen. Prior to the pandemic, a business healthcare management team (aag.nl) developed a plan of demands to inform the design of a newly built psychiatric hospital for the GGZ in Leeuwarden. The development plan was based on the centralization of inpatient and outpatient services. The plan detailed the needs of the GGZ staff and patients based on a detailed consultative and participatory design process.

The design and the architecture of this new psychiatric hospital, inclusive of 72 inpatient beds and an outpatient facility, is a collaborative effort consisting of GGZ Friesland staff project committee, a patient advisory committee, and an architecture firm (Adema Architecten). The collaborative design process is led by architects, designers, and a researcher who consult with service users (i.e., staff, patients, and other service providers in the treatment system) with experience using GGZ facilities. During monthly design discussions, 2-D and 3-D virtual mock-ups are interactively presented online. The design team provides a detailed overview of the design and a summary of evidence-based design decisions. Service users are invited to provide feedback through discussions, chat functions, or digital workspaces created with the software Mural. The evidence-based design approach to inform design decisions is based on interdisciplinary scientific research, which links design and environmental features to patient and staff outcomes inspired by several research studies including work by Shepley and Pasha (2017), Shepley et al. (2016), and Ulrich et al. (1991, 2018, 2020). In particular, a research focus on design interventions that prove to reduce stress and aggression and improve the psychiatric environment’s safety was translated into the new design (Shepley et al., 2016; Ulrich et al., 2018). For the new design of the psychiatric environment, the project team referred to Ulrich et al.’s (2018) theory that states hospitals can be safer and more healing for patients through a range of interventions, including single-bed rooms, effective ventilation systems, a good acoustic environment, nature distractions and daylight, appropriate lighting, better ergonomic design, acuity-adaptable rooms, and improved floor layouts and work settings. Similarly, other design features such as well-integrated facilities with smaller, more home-like units, single or double bedrooms, and a wide range of communal areas combined with open nursing stations can create a good balance between private and shared space (Jovanović et al., 2019; Shepley et al., 2016). Further, the design reflects the concept of positive health, which presents a new view of health with a focus on the ability of individuals to adapt and self-manage when experiencing social, physical, and emotional challenges. The concept has a range of indicators categorized into six domains: bodily functions, mental functions and perception, spiritual/existential, quality of life, social and societal participation (social health), and daily functioning (Huber et al., 2011; Huber et al., 2016). Positive health is embedded in the corporate structure of the GGZ and reflected several health policies, including built environment initiatives in the Netherlands (Den Broeder et al., 2017; Steensma, 2021).

***Further, the design reflects the concept of positive health, which presents a new view of health with a focus on the ability of individuals to adapt and self-manage when experiencing social, physical, and emotional challenges.***

## Findings

The therapeutic psychiatric environment in Friesland changed significantly during COVID-19. The number of patients accessing care decreased, patient autonomy was restricted, the function of designated behavioral support spaces changed to manage the risk of infection, and new forms of service provision emerged. These descriptions and reflections provide insight into how to design new psychiatric environments.

***The therapeutic psychiatric environment in Friesland changed significantly during COVID-19. The number of patients accessing care decreased, patient autonomy was restricted, the function of designated behavioral support spaces changed to manage the risk of infection, and new forms of service provision emerged.***

## Descriptions and Reflections on Inpatient Psychiatric Care

### Occupancy Rates and Critical Incidents

Compared to previous years, there were fewer admitted patients across the two psychiatric sites during the pandemic. Fewer patients impacted the social environment, a central component of the psychiatric treatment environment. There were 26% fewer intakes, and the length of stay decreased by 21%. The psychiatrist noted that particularly patients admitted for depression decided to discharge themselves, where staying confined to a bedroom on the ward with limited nature views was not helping with the depressive symptoms. The decrease in psychiatric hospital admissions was also noted in other sites internationally (Beghi et al., 2021; Gómez-Ramiro et al., 2021; Pignon et al., 2020). Further, similar to other countries, there were fewer involuntary admissions compared to 2019 (Chen et al., 2020; Gómez-Ramiro et al., 2021). However, the number of critical incidents involving aggression increased from 80 incidents in total in 2019 to 125 in total in 2020, and as of September 2021, there were already 101 critical incidents recorded.

### Adapting the Use of the Physical Environment to Manage Infection

Throughout the pandemic, the physical environment was modified to control infection risk and continue patient care. To limit the risk of infection for patients and staff, alcohol-based hand-rub dispensers were installed at the entrance of the ward and flasks with alcohol-based hand-rub were placed on the ward itself in supervised locations (i.e., nurse station, speaking room). When patients arrived in the ward, they were tested in their rooms. If there was a suspected or confirmed COVID-19 infection on the ward, positive cases were transferred to one of the few isolation rooms. Isolation rooms in psychiatry usually are only used for emergencies to isolate patients who are a physical threat to themselves or others. However, where psychiatric environments are not typically designed to manage infection, these rooms were deemed the most suitable spaces to quarantine patients. Other psychiatric care studies have also described the movement of positive cases to isolation units (Kreuzer et al., 2020). The use of these rooms was modified by keeping the doors open and allowing patients with space to move around. In circumstances where patients would refuse testing or wear masks, they were also transferred to the isolation environment during the 5-days incubation period of the virus. There are a limited number of isolation rooms. As the infection rates intensified, additional rooms were designated in the ward to manage confirmed or suspected infections.

**Figure 2.**
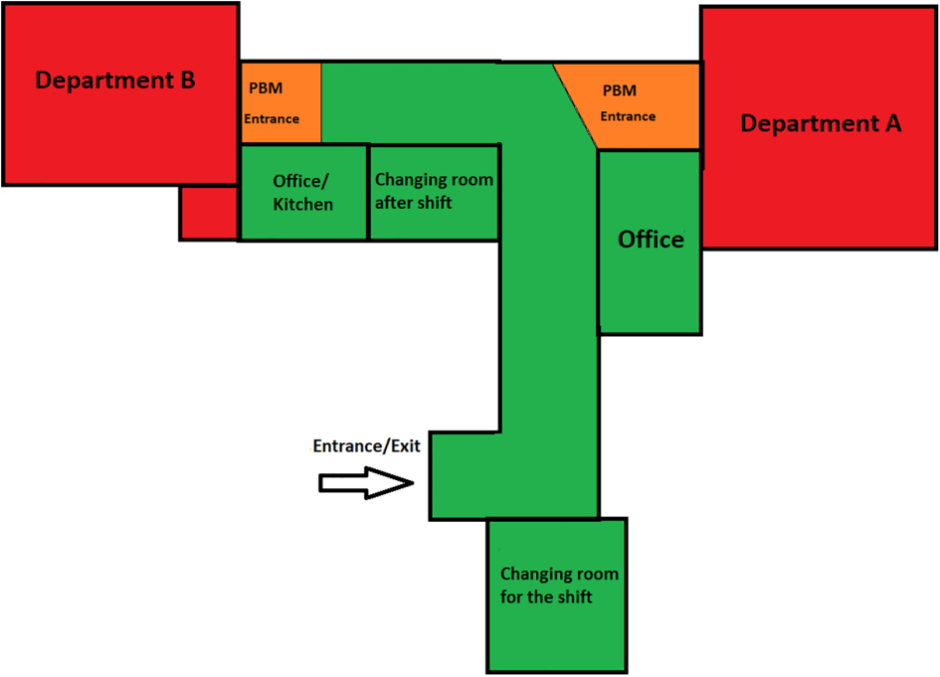
Preventative zoning and staff routing map.

The physical environment was further modified by adding a “lock” (i.e., a section bounded by two doors), making it possible to create an isolated environment for up to three patients. As seen in Figure 2, preventative zones (e.g., the orange zones) were created to minimize the risk of staff bringing COVID-19 to the ward. To manage the risk of infection, staff came on to the ward through the preventative zone, where they could change into protective clothing for their shift and then after their shift. There was a separate space designated to change out of protective clothing. However, wearing personal protective equipment (PPE) at times (i.e., during a violent escalation on the ward) made it unsafe for staff, especially when administering mandatory intramuscular injection medication. In some cases, staff took a risk and did not wear all the PPE, for instance, aprons. Therefore, as the pandemic continued, the purpose of preventative zoning was used less often.

### Impact on Patient Autonomy and Therapeutic Encounters

Due to the high risk of infection, no visitors was permitted on the ward, which impacted the social component of the therapeutic landscape of the psychiatric ward. Further, patient autonomy and interaction were restricted, with most patients confined to their rooms with meals and medication delivered by the nurses. Changing this level of interaction impacted the therapeutic environment, where patients are typically encouraged to leave their rooms, go to the nursing station for medication, and eat together in a communal setting. Further, to limit cross-contamination, patients were kept separate from one another at all times, and patient autonomy was entirely dependent on nurses who had to escort individual patients to the toilet, shower, and smoking breaks. The nurses relocated the nursing station to better respond to patients’ requests (e.g., a table). They placed it outside the patient rooms allowing for better patient observation and to keep up with patients’ requests. This make-shift nursing station was not ideal for the patients or the staff. There were also fewer therapeutic activities both on-site and outside the hospital. Due to the restrictions, doing a trial home visit was not possible, which was typical for patients near the end of their treatment. In the later months, it was decided to allow the patients more autonomy to move around the ward independently and interact with one another.

### Digital Transformation of Service Provision

Similar to most work environments throughout the pandemic, when possible, GGZ staff were encouraged to work from home as much as possible, replacing in-person meetings with video and telemedicine devices (Lyne et al., 2020; Naarding et al., 2020). Patients could use their phones and devices to stay virtually in contact with friends and family. Similar to other studies, before COVID-19, telecommunication and telepsychiatry were not typical in psychiatric wards (Khanna & Forbes, 2020). However, the use of technology during the pandemic enhanced the otherwise restricted therapeutic environment. As the pandemic prolonged, video conferencing was used more often for patient care and decision making. In Friesland, this digital transformation started with laptops and video conferencing for the psychiatrist, who was in quarantine, to communicate with a patient in seclusion. The video allowed the psychiatrist to assess patients remotely. As the pandemic prolonged, video conferencing made the clinical practice more efficient for some professionals, but it was not as effective for speakerphone conversations.

Similarly, as noted in other psychiatric contexts (Kennedy et al., 2020), patient court hearings took place by speakerphone, not video. For many months, communication with the patient, their lawyers, judges, and the psychiatrist was restricted through this type of media. Not having a visual of the patient made it challenging to make clinical decisions. These situations improved with a video conferencing system. The video conferencing system Teams was introduced by the GGZ. Portable Teams screens and designated conference rooms with Teams installed changed and enhanced the therapeutic environment during the pandemic—this digital environment allowed for meetings with the patients and health professionals. Further, two rooms were designated for Teams communication.

Most notable was the role Teams played in supporting the work of the function assertive community treatment (FACT) team. FACT teams are a part of the GGZ psychiatry model. FACT is the Dutch evolved version of the assertive community team model and is an interdisciplinary, individual-based case management approach to providing a continuity of care to patients with serve mental illness who live in the community but have histories being admitted for inpatient psychiatric care (Drukker et al., 2008). In Friesland, the FACT team works across the province and in rural and remote locations. Like other sites globally, the FACT team had to adapt and modify their case management in response to COVID-19 restrictions (Couser et al., 2021; Guan et al., 2021; Law et al., 2021). The digital platform of Teams allowed the FACT team to have frequent contact with the hospital staff and saved time on travel. The adaption of the environment and the use of Teams will likely continue in the practice where video conferencing saves travel time for staff outside the ward and is seen as an effective way to make remote decision making in psychiatric care (Best & Ingram, 2020; Ingram & Best, 2020).

## Preliminary Design of New Psychiatric Environment

**Figure 3.**
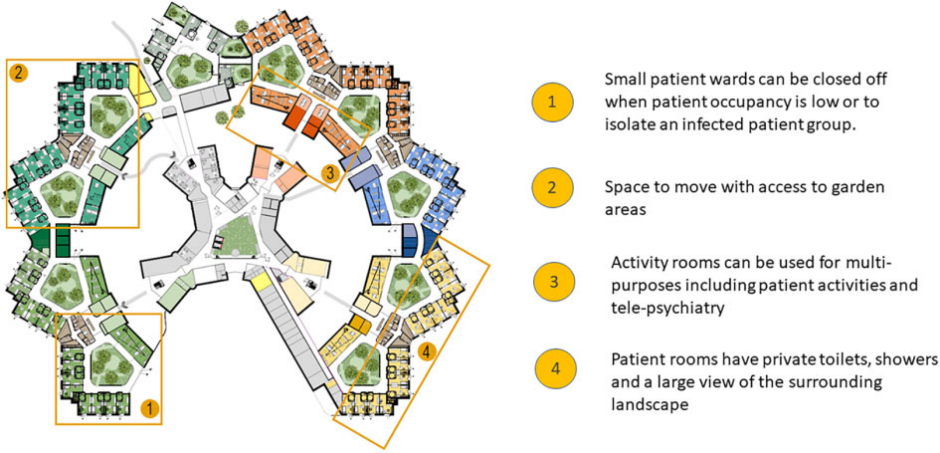
Preliminary design of a new psychiatric environment (illustrated by Gerben van der Heide, Adema Architecten).

**Figure 4.**
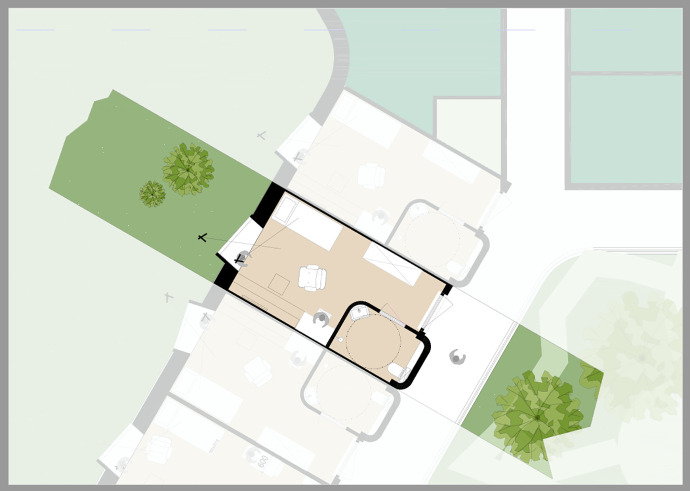
Preliminary design of a patient room (illustrated by Gerben van der Heide, Adema Architecten).

A project team from the GGZ and the architecture team met biweekly, online to make collaborative design decisions throughout the pandemic. These design decisions were based on different levels of experience, plan of demands, research, best practices, and clinical expertise. Participating in these discussions during the pandemic provided an opportunity to reflect and ensure that the new design would provide continuity of care during a pandemic such as COVID-19. For instance, the current environments are based on a corridor-based floor plan, and patients share toilets and showers and have limited access to garden areas. As described in the descriptions above, the current environment made it difficult to continue care during the pandemic, which impacted patient treatment. As seen in Figure 3, the new design would better support patient well-being, autonomy, and a continuation of care in circumstances of a pandemic similar to COVID-19. The preliminary design is adaptable based on a centralized ward layout with patient rooms organized around a central area with access to unlocked garden areas (Ulrich et al., 2018, 2020). The new layout reduces patient crowding through single-patient rooms (Figure 4) with private showers and toilets and views of outdoor environments (i.e., green landscapes). Even as patients would be restricted to the ward, the new design allows patients to walk around the garden area, access the gardens, and have garden views, which are considered a positive distraction to reduce patient stress (Ulrich et al., 2018). Further, the adaptive design and flexible layout allow the hospital administrator to adapt the use of the site based on patient or operational needs. This design is especially relevant to psychiatric care, where it is expected that inpatient psychiatry will advance over time primarily through the use of technology. Therefore, when the patient occupancy rates are low on one ward, the new design allows patients to relocate to another ward allowing for more flexible capacity, including staffing efficiency.

## Discussion

The COVID-19 pandemic impacted and halted healthcare systems worldwide. COVID measures had a noticeable impact on the relational dynamics of psychiatric wards, including a lack of activities and visitors, which others have identified as an essential aspect of the therapeutic landscape related to well-being (Duff, 2012; Moon et al., 2016). In general, there is limited research on how the COVID-19 pandemic impacted the inpatient psychiatry environment (Baumgart et al., 2021). This case study contributes to this emerging area of research by detailing how the inpatient psychiatric environment was restructured in Friesland, the Netherlands, to manage the infection and continue to provide clinical support. The reflections made in this study are similar to other international psychiatric studies that noted that psychiatric services became more flexible and practical to respond to COVID-19 (Barry et al., 2020). Even more, reflecting on the modifications to the built and social environment made during the pandemic provides an opportunity to design psychiatric environments for future pandemics or other demands for healthcare (Honey-Rosés et al., 2020; Shepley et al., 2021). Adaptive, flexible design strategies for hospitals and designing for unknown functions is not commonly researched (Pilosof, 2021). Based on this case study and similar to other studies, future research should explore how the built environment can respond to future functions and changes in the delivery of healthcare. The flexible layout of the preliminary design, based on theories, would have been useful during the pandemic to mitigate the risk of transmission, especially when staff availability was at times limited. The organization of the preliminary design would allow for other types of healthcare services to be easily set up in this environment, such as geriatric care, or use the site for a different purpose, such as long-term supportive housing.

As described in this study, the COVID-19 pandemic presented challenges to keep patients and staff safe from infection and provide a therapeutic environment. Prior to the pandemic, providing a healing and safe environment for patients was already said to be challenging when designing psychiatric wards (Lundin, 2021). Therefore, designing a new psychiatric hospital during COVID-19 allowed to translate tactic knowledge to be reflected in the design. Despite the nuance of COVID-19, earlier theories, such as Florence Nightingale’s environmental theory, relate to designing hospitals that remain relevant in light of the pandemic (Fernandes & Silva, 2020; Gilbert, 2020). As the theory states, all hospitals should have pure air, pure water, efficient drainage, cleanliness, and light. Further, other theories, such as the stress reduction theory, note that a healing environment should have adequate ventilation systems, nature distractions and daylight, adaptable rooms, and improved floor layouts (Ulrich, 1991; Ulrich et al., 1991, 2018). Not only are these theories foundational for providing quality care and reducing patient and staff stress, but these theories can also be related to designing renovating care environments to manage infections, such as COVID-19 and continuing care.

This article attempts to link clinical reflections to the concept of therapeutic landscapes by reflecting on how the therapeutic landscape, including describing how the setting, locales, and milieus, changed in response to COVID-19. A focus on place and space provides contextual insight into how an environment was used and modified to mitigate the risk of infection. As described, patients’ well-being was impacted when their level of autonomy was restricted with minimal social interaction or engagement with blue and green landscapes. However, this study describes a new milieu in the therapeutic landscape. During this time, a digital landscape emerged and supported the continuity of patient care/and therapeutic landscape. This new therapeutic landscape, created with the Teams environment, created a health-enabling, digital environment (i.e., telepsychiatry) that appears to contribute to the therapeutic environment positively. Unlike the blue or green landscapes, which are nature-based, the digital landscape is a virtual landscape, which allows patients to engage in another dimension of “place,” care, connection, and healing. According to other research, most patients were satisfied with the digital remote care and assessment format during the pandemic (Li et al., 2021; Soron et al., 2020). However, this type of landscape that will promote patient autonomy on a longer term basis has not been explored (Columb et al., 2020; Sasangohar et al., 2020). Concerning psychiatric therapeutic landscapes, digital landscapes should be explored to understand how patient healing works in these digital places.

***Concerning psychiatric therapeutic landscapes, digital landscapes should be explored to understand how patient healing works in these digital places.***

### Strengths and Limitations

The strength of this case study is the transdisciplinary approach to design during the COVID-19 pandemic. Architecture, geography, and psychiatry are vastly different disciplines yet, and they have a common interest in the interaction of people with their environment and are often related to cultural and societal trends (Shorter, 2008). Despite these similarities, the health perspectives of geographers, architects, and psychiatrists are rarely reflected in the design phase of healthcare architecture. However, the knowledge between these disciplines has long been implicit in hospital design. For instance, Nightingale’s early work emphasized the importance of considering the individual (the patient) in the interaction with the environment (geography) to design environments (architecture) that support the best possible conditions for healing to occur. A transdisciplinary approach provides a unique perspective for designing healthcare, which can guide design decisions and reflect the direct experiences of patients and staff who use the facilities (Pink et al., 2020; Shepley et al., 2016). This transdisciplinary approach provides an opportunity to link credible research across disciplines, enriching the design and creating indicators for the best possible outcomes to support evidence-based design.

***This transdisciplinary approach provides an opportunity to link credible research across disciplines, enriching the design and creating indicators for the best possible outcomes to support evidence-based design.***

Similar to other COVID-19 studies (Couser et al., 2021; Guan et al., 2021; Law et al., 2021), this article is not based on empirical data. This study is theoretical and reflective based on clinical reflections over the past 18 months. Moreover, the descriptions in this study do not include the perspective of the staff or patients who were in these environments during different phases of the pandemic. Further, this study does not evaluate the impact of collaboration on predesign. Like Jouppila (2021), future research should systematically explore and evaluate the collaborative participation of this project to provide a more detailed impression of how their impression of the clinical environment changed during the pandemic and how new healthcare environments can be designed to be more responsive.

## Conclusion

Psychiatric environments are often designed based on safety and protection from self-harm without a dedicated focus on infection control. During the pandemic, the psychiatric environment in Friesland was restructured to ensure the continuity of care for patients and limit the number of COVID-19 infections. This transdisciplinary study describes some of these modifications made to the environment. Based on these reflections, design discussions, and an integrated theoretical approach to design, a preliminary design of a new psychiatric environment is presented. This study suggests that COVID-19 provided an opportunity to reflect on how psychiatric environment are built to be more adaptable to manage infection and continue care. The science remains unclear how long the pandemic will last or the long-term impact on physical and mental health. Designing environments informed by theory to create hospital environments to more healing and safe, and meanwhile reflecting on knowledge created during the pandemic, will allow for therapeutic environments to be designed to adapt to new service needs and better respond to further healthcare crises.

***Designing environments informed by theory to create hospital environments to more healing and safe, and meanwhile reflecting on knowledge created during the pandemic, will allow for therapeutic environments to be designed to adapt to new service needs and better respond to further healthcare crises.***

## Implications for Practice

The inpatient psychiatric environment was restructured at GGZ Friesland in the Netherlands to manage the infection and continue to provide clinical support.

- An interprofessional study provides both theoretical reflection and clinical reflections into the inpatient psychiatric care environment.
- A digital landscape supports the continuity of patient care/and therapeutic landscape of a psychiatric environment.
- Design decisions were based on different levels of experience, plan of demands, research, best practices, and clinical reflections and descriptions.

Reflecting on the modifications to the built and social environment made during the pandemic provides an opportunity to design psychiatric environments for future pandemics or other demands for healthcare.
